# Supplementary material for: Single-molecule analysis reveals cooperative stimulation of Rad51 filament nucleation and growth by mediator proteins
Source: Mol Cell. 2021 Mar 4;81(5):1058–1073.e7. doi: 10.1016/j.molcel.2020.12.020 (PMC7941204; doi:10.1016/j.molcel.2020.12.020)
Supplement: Document S1. Figures S1–S7 and Table S1 [file mmc1.pdf]

Molecular Cell, Volume *81*

Supplemental information

Single-molecule analysis reveals cooperative stimulation of Rad51 filament nucleation and growth by mediator proteins

Ondrej Belan, Consuelo Barroso, Artur Kaczmarczyk, Roopesh Anand, Stefania Federico, Nicola O'Reilly, Matthew D. Newton, Erik Maeots, Radoslav I. Enchev, Enrique Martinez-Perez, David S. Rueda, and Simon J. Boulton

Figure S1

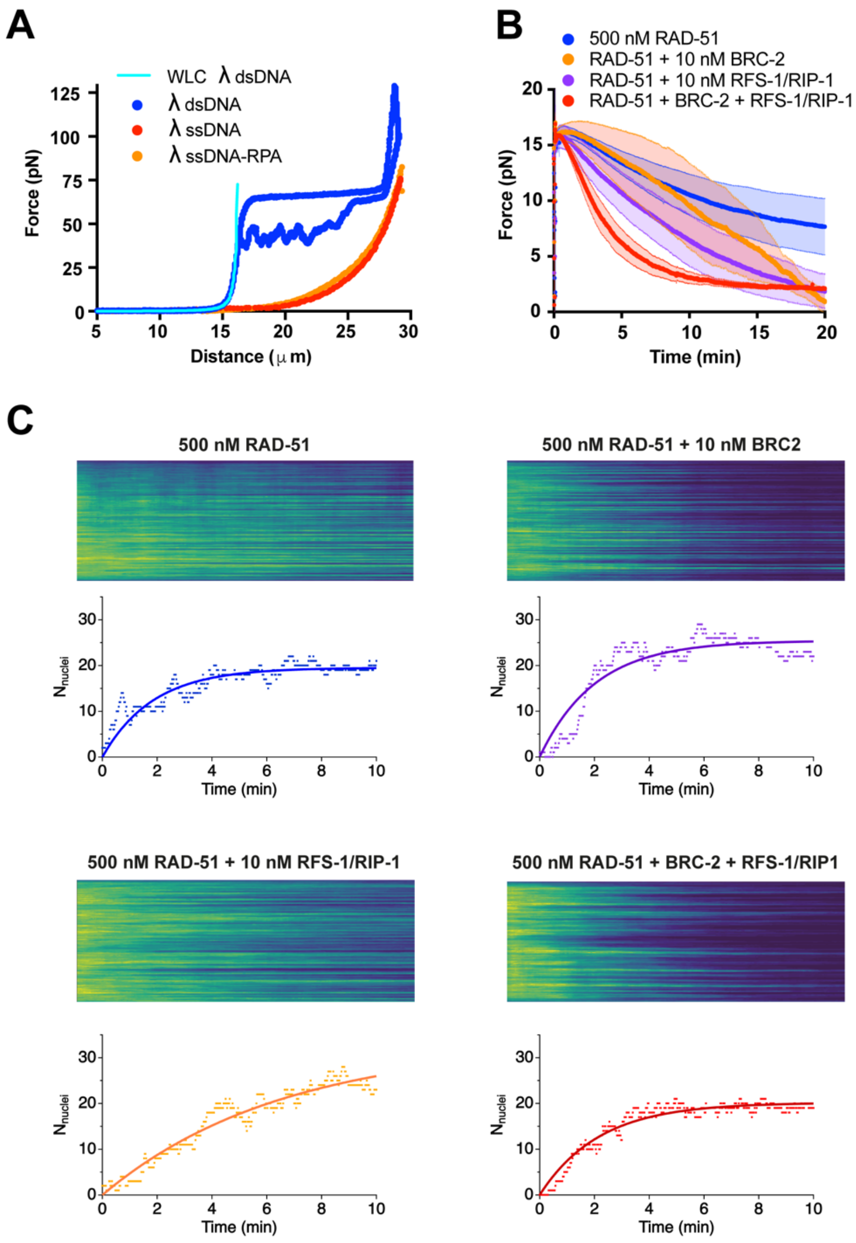

A
WLC λ dsDNA
λ dsDNA
λ ssDNA
λ ssDNA-RPA
Force (pN)
Distance (μ m)
B
500 nM RAD-51
RAD-51 + 10 nM BRC-2
RAD-51 + 10 nM RFS-1/RIP-1
RAD-51 + BRC-2 + RFS-1/RIP-1
Force (pN)
Time (min)
C
500 nM RAD-51
500 nM RAD-51 + 10 nM BRC2
500 nM RAD-51 + 10 nM RFS-1/RIP-1
500 nM RAD-51 + BRC-2 + RFS-1/RIP1
N nuclei
Time (min)

Figure S1. Force-extension curves recorded for different substrates and additional kymograph analysis. Related to Fig. 1. (A) Force extension curves of 48.5 kb λ dsDNA (forward and reverse curve after partial melting), 48.5 kb λ ssDNA and 48.5 kb λ ssDNA coated by RPA. Blue line represents worm-like chain model (WLC) fit as a reference. RPA addition does not substantially change the shape of force-extension curve of λ ssDNA. **(B)** Force measured between the traps as a function of time in the indicated conditions; shaded area represents SEM. (n = 3-8 molecules). **(C)** Quantification of RAD-51 nucleation frequency over time on representative single molecules of 48.5 kb λ ssDNA in the absence of mediator proteins (upper left panel), in the presence of 10 nM BRC-2 (upper right panel), in the presence of 10 nM RFS-1/RIP-1 (lower left panel), and in the presence of both mediators (lower right panel). Number of nuclei was calculated for each time frame of the median-filtered and smoothed kymograph by detecting peaks in the reversed intensity profile. Black lines represent the exponential fit to the first 400 frames (10 min).

Figure S2

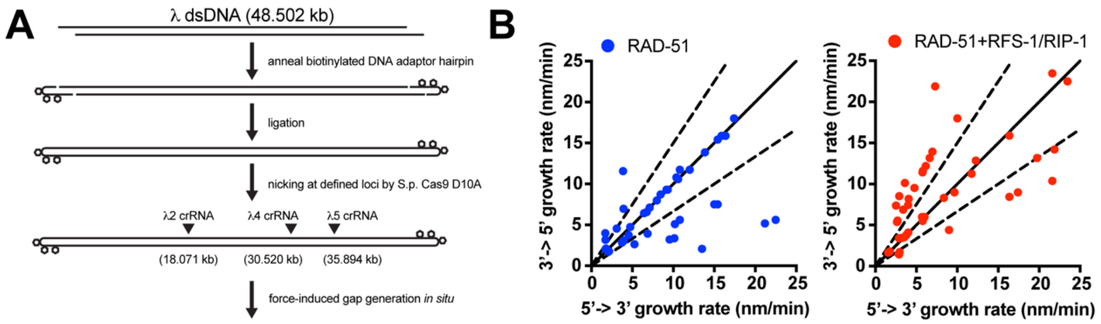

Figure S2. Generation of gapped DNA (gDNA) substrates and filament growth analysis. Related to Fig. 3. (A) A schematic of protocol designed to generate gapped λ DNA (gDNA) substrate. Two pairs of guide RNAs (λ4 crRNA and λ5 crRNA; λ2 crRNA and λ5 crRNA) and subsequent force-induced DNA melting *in situ* were utilized to generate 5.3 and 17.8 kb ssDNA gaps. **(B)** Scatterplot of 5' to 3' and 3' to 5' growth rates of individual RAD-51 clusters in the absence or presence of 10 nM RFS-1/RIP-1 using λ gDNA substrates. 10 nM free RPA-eGFP was included in a fraction of reactions performed and analysed to suppress excessive nucleation and obtain better-resolved growing clusters. Full line represents linear regression function with slope = 1, dotted lines represent linear regression function with slope = 1.5 or slope = 0.67.

Figure S3

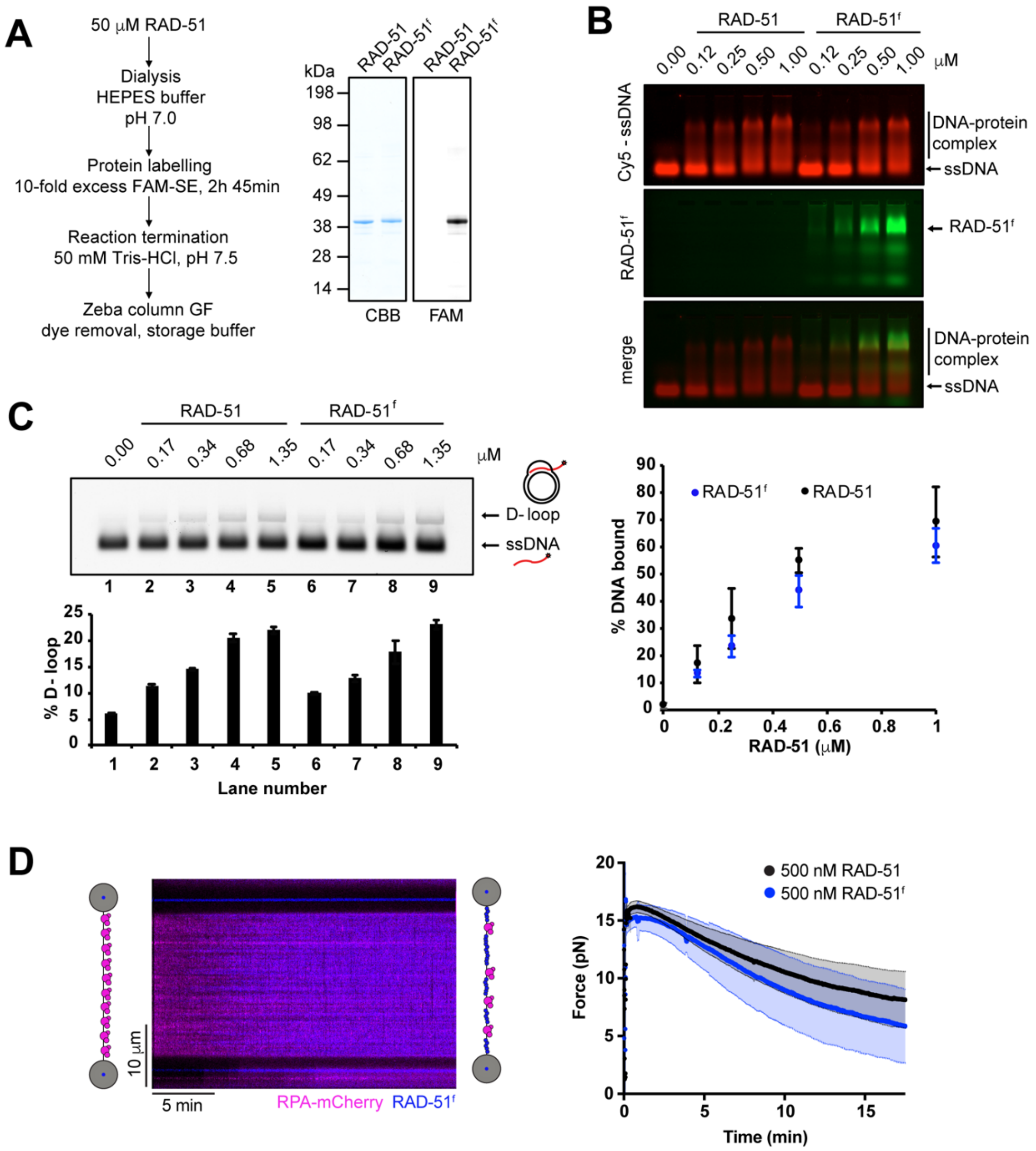

A
50 µM RAD-51
Dialysis
HEPES buffer
pH 7.0
Protein labelling
10-fold excess FAM-SE, 2h 45min
Reaction termination
50 mM Tris-HCl, pH 7.5
Zeba column GF
dye removal, storage buffer
kDa
198
98
62
49
38
28
14
RAD-51
RAD-51f
CBB
FAM
B
RAD-51
RAD-51f
0.00 0.12 0.25 0.50 1.00 0.12 0.25 0.50 1.00 µM
Cy5 - ssDNA
RAD-51f
merge
DNA-protein complex
ssDNA
C
0.00 0.17 0.34 0.68 1.35 0.17 0.34 0.68 1.35 µM
D- loop
ssDNA
1 2 3 4 5 6 7 8 9
% D - loop
Lane number
RAD-51f
% DNA bound
RAD-51 (µM)
D
10 µm
5 min
RPA-mCherry
500 nM RAD-51
500 nM RAD-51f
Force (pN)
Time (min)

Figure S3. Fluorescent labelling of RAD-51. Related to Fig. 4. (A) Chemical labelling of RAD-51. RAD-51 was labelled in pH 7.0 using succinyl esters of 6-FAM (FAM-SE). After reaction termination and purification of labelled species, labelling efficiency was assessed and free dye component was evaluated using SDS-PAGE and subsequent fluorescent imaging. 1:1 labelling stoichiometry was achieved as measured spectrophotometrically. Higher labelling ratios attenuate RAD-51 DNA binding and D-loop formation activities. Proteins were labelled typically with 80-100% labelling efficiency. **(B)** EMSA showing that both RAD-51 and RAD-51^{f} bind ssDNA to a similar extent. Proteins were incubated with 20 nM (in molecules) FAM-labelled 49-mer ssDNA for 10 minute at 25 °C. DNA-protein complexes were resolved for 60 min at 70V in 0.8% agarose gel TAE electrophoresis at 4 °C. (n = 3 replicates). Error bars represent SD. **(C)** D-loop formation assay comparing DNA-pairing activities of RAD-51 and RAD-51^{f} . Proteins were incubated with 30 nM (in molecules) FAM-labelled 90-mer ssDNA for 10 minute at 25 °C followed by addition of 0.54 μg pBS(-) plasmid DNA. Reactions were terminated by SDS-Proteinase K treatment for 10 min at 37 °C. DNA was resolved for 30 min at 90V in 0.8% agarose gel TAE electrophoresis at 4 °C. (n = 3 replicates). Error bars represent SD. **(D)** Kymograph showing the displacement of RPA-mCherry by 500 nM RAD-51^{f}. Force measured between the traps as a function of time for 500 nM RAD-51 assembly on RPA-eGFP-coated ssDNA and 500 nM RAD-51^{f} assembly on RPA-mCherry-coated ssDNA; shaded area represents SEM. (n = 5-8 molecules).

Figure S4

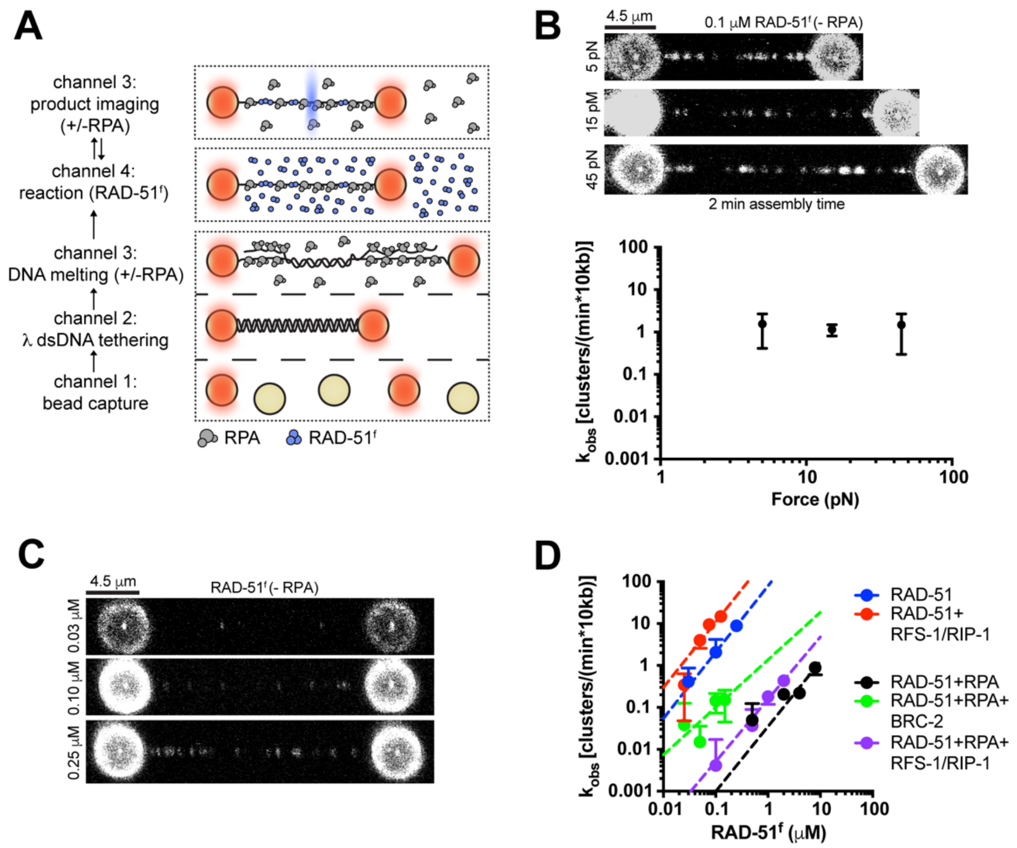

Figure S4. 'Dipping' protocol to determine RAD-51 nucleation rates. Related to Fig. 4. (A) A four-channel microfluidics device was used for the assembly of the 5-step single-molecule binding experiments: (1) trapping of streptavidin-coated microspheres and (2) tethering of a single λ dsDNA between the spheres, (3) force-induced melting of dsDNA into ssDNA, (4) incubation in a flow channel with fluorescent RAD-51^f (blue) and ATP, and visualization of the RAD-51^f DNA complex in observation channel and buffer containing ATP. **(B)** Representative fluorescence images of RAD-51^f clusters formed on ssDNA molecules held at 5, 15 and 45 pN force. 4.5 μm scale bar. RAD-51^f nucleation rates at 5, 15 and 45 pN forces are plotted. **(C)** Representative fluorescence images taken after 30s of RAD-51^f incubation–detection cycle with different RAD-51^f concentrations present in the solution. 4.5 μm scale bar. **(D)** RAD-51^f concentration dependence of nucleation rate. Dotted line represents power-law fit ($k_{obs} = J[\text{RAD-51}]^n$) yielding an exponent n = 1.6 ± 0.2 for RAD-51^f in the absence of RPA (R^2 = 0.72), n = 1.6 ± 0.2 for RAD-51^f in the absence of RPA and the presence of stoichiometric amounts of RFS-1/RIP-1 (R^2 = 0.90), n = 1.6 ± 0.4 for RAD-51^f in the presence of RPA (R^2 = 0.83), n = 1.1 ± 0.4 for RAD-51^f in the presence of RPA and stoichiometric amounts of BRC-2 (R^2 = 0.21) and n =1.5 ± 0.1 for RAD-51^f in the presence of RPA and stoichiometric amounts of RFS-1/RIP-1 (R^2 = 0.88). Error bars indicate SD.

Figure S5

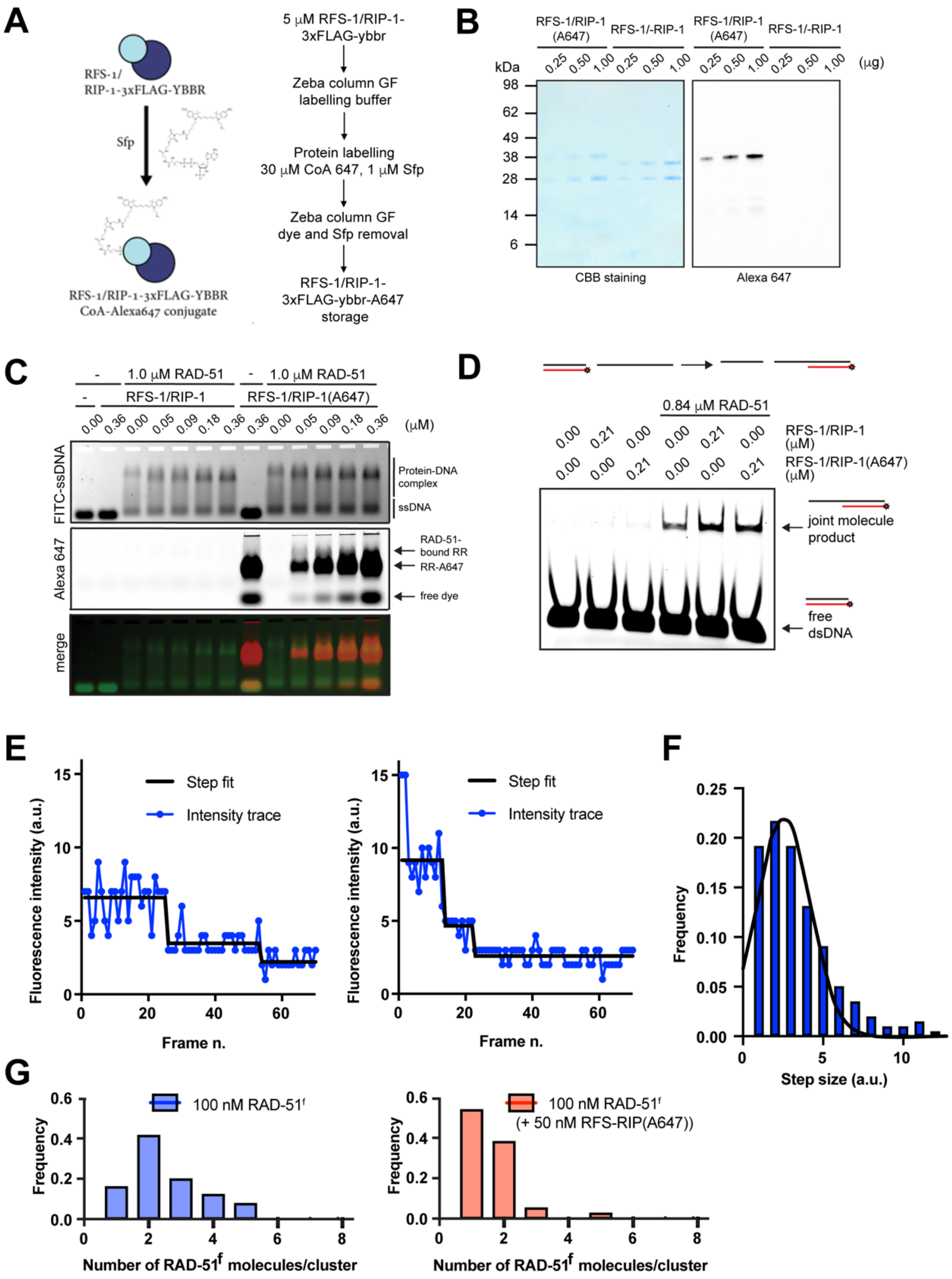

A
RFS-1/RIP-1-3xFLAG-YBBR
Sfp
RFS-1/RIP-1-3xFLAG-YBBR CoA-Alexa647 conjugate
5 μM RFS-1/RIP-1-3xFLAG-ybbr
Zeba column GF labelling buffer
Protein labelling 30 μM CoA 647, 1 μM Sfp
Zeba column GF dye and Sfp removal
RFS-1/RIP-1-3xFLAG-ybbr-A647 storage
B
RFS-1/RIP-1 (A647)
RFS-1/-RIP-1
(μg)
kDa
CBB staining
Alexa 647
C
1.0 μM RAD-51
RFS-1/RIP-1
RFS-1/RIP-1(A647)
(μM)
FITC-ssDNA
Alexa 647
merge
Protein-DNA complex
ssDNA
RAD-51-bound RR
RR-A647
free dye
D
0.84 μM RAD-51
RFS-1/RIP-1 (μM)
RFS-1/RIP-1(A647) (μM)
joint molecule product
free dsDNA
E
Step fit
Intensity trace
Fluorescence intensity (a.u.)
Frame n.
F
Frequency
Step size (a.u.)
G
100 nM RAD-51f
100 nM RAD-51f (+ 50 nM RFS-RIP(A647))
Frequency
Number of RAD-51f molecules/cluster

Figure S5. Fluorescent labelling of RFS-1/RIP-1 and single-step photobleaching analysis. Related to Fig. 4. **(A)** Scheme and protocol for fluorescent labelling of ybbr-tagged RFS-1/RIP-1 complex. **(B)** Coomasie Brilliant Blue staining (left) and A647 fluorescence (right) of SDS-PAGE resolved RFS-1/RIP-1-3xFLAG or RFS-1/RIP-1-3xFLAG-ybbr-CoA-Alexa647 proteins. Loaded amount of protein is indicated. Proteins were labelled typically with 70-80% labelling efficiency. **(C)** EMSA showing that both RFS-1/RIP-1-3xFLAG and RFS-1/RIP-1-3xFLAG-ybbr-CoA 647 bind RAD-51-ssDNA complexes and induce a similar electrophoretic mobility shift. Proteins were incubated with 30 nM (in molecules) FAM-labelled 49-mer ssDNA for 10 min at 25 °C in the absence of glutaraldehyde crosslinking. DNA-protein complexes were resolved for 60 min at 70V in 0.8% agarose gel 1x TAE electrophoresis at 4 °C. **(D)** Strand exchange assay. Proteins were incubated for 10 min with 5.6 nM (in molecules) 150-mer oligonucleotide at 25 °C for 10 min. 8 nM (in molecules) dsDNA stock and 4 mM spermidine were then added followed by further incubation for 1.5 h at 25 °C. The samples were deproteinized with 0.1% SDS and 12.5 μg proteinase K at 37 °C and resolved using PAGE in 1x TBE. **(E)** Examples of a fluorescence intensity trace of the RAD-51^f cluster during continuous photobleaching. Black line represents stepping fit of the trace. **(F)** Fluorescence intensity single-molecule calibration histogram for RAD-51^f. The histogram shows the values of the fluorescence intensity trace of the single steps of multiple photobleaching traces (n = 198 steps). Black line represents gaussian fit. Second two-step gaussian was excluded from the analysis. Mean = 2.55. S.D. = 1.67. R^2 = 0.90. **(G)** Histogram of RAD-51^f cluster size in the presence (n = 76 clusters) or absence (n = 181 clusters) of RFS-1/RIP-1(A647).

Figure S6

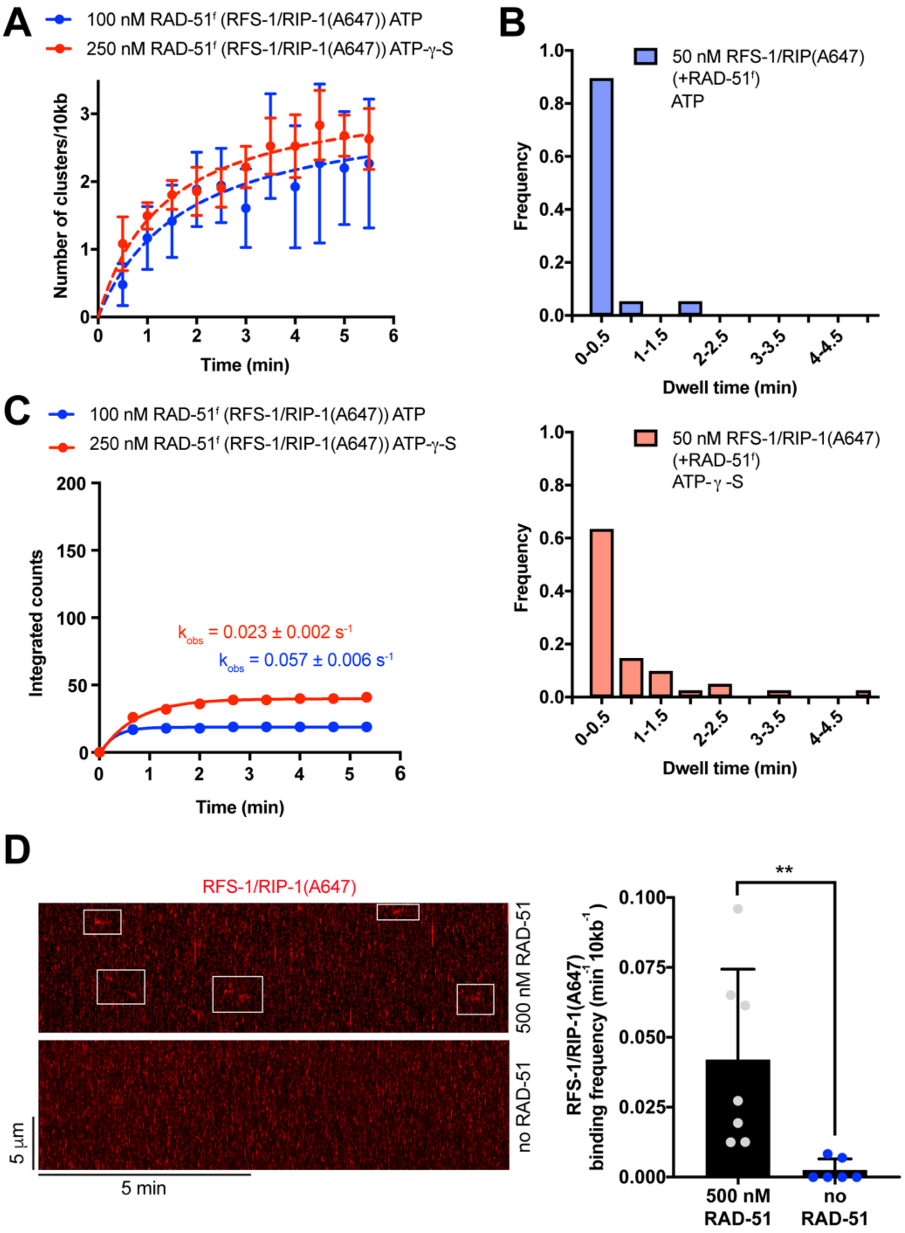

A
100 nM RAD-51f (RFS-1/RIP-1(A647)) ATP
250 nM RAD-51f (RFS-1/RIP-1(A647)) ATP-γ-S
Number of clusters/10kb
Time (min)
B
50 nM RFS-1/RIP(A647) (+RAD-51f) ATP
Frequency
Dwell time (min)
50 nM RFS-1/RIP-1(A647) (+RAD-51f) ATP-γ -S
C
100 nM RAD-51f (RFS-1/RIP-1(A647)) ATP
250 nM RAD-51f (RFS-1/RIP-1(A647)) ATP-γ-S
Integrated counts
kobs = 0.023 ± 0.002 s-1
kobs = 0.057 ± 0.006 s-1
Time (min)
D
RFS-1/RIP-1(A647)
500 nM RAD-51
no RAD-51
5 μm
5 min
RFS-1/RIP-1(A647) binding frequency (min-1 10kb-1)
**
500 nM RAD-51
no RAD-51

Figure S6. RFS-1/RIP-1(A647) dwell-times in the presence of ATP-γ-S. Related to Fig. 5. (A) Quantification of RAD-51^{f} nucleation frequency over time in the presence of ATP (n = 9 molecules) or ATP-γ-S (n = 9 molecules) and RFS-1/RIP-1(A647) in the absence of RPA. Exponential fits are displayed as dashed lines. Error bars represent SEM. RAD-51 concentrations were chosen to yield similar nucleation frequencies. In similar experiment, negligible nucleation frequencies were observed with 100 nM RAD-51 alone. **(B)** Histograms of dwell times of RFS-1/RIP-1(A647) in the presence of RAD-51^{f} with ATP or ATP-γ-S. **(C)** Cumulative survival plots of data presented in Fig S7B. Lines represent exponential fits. **(D)** Representative kymographs of dwelling single RFS-1/RIP-1(A647) (2.5 nM) complexes on RPA-eGFP coated ssDNA in the presence or absence of 500 nM RAD-51 and ATP. Quantification of RFS-1/RIP-1(A647) binding frequencies on individual DNA molecules is shown on the left. p = 0.0012. Mann-Whitney test.

Figure S7

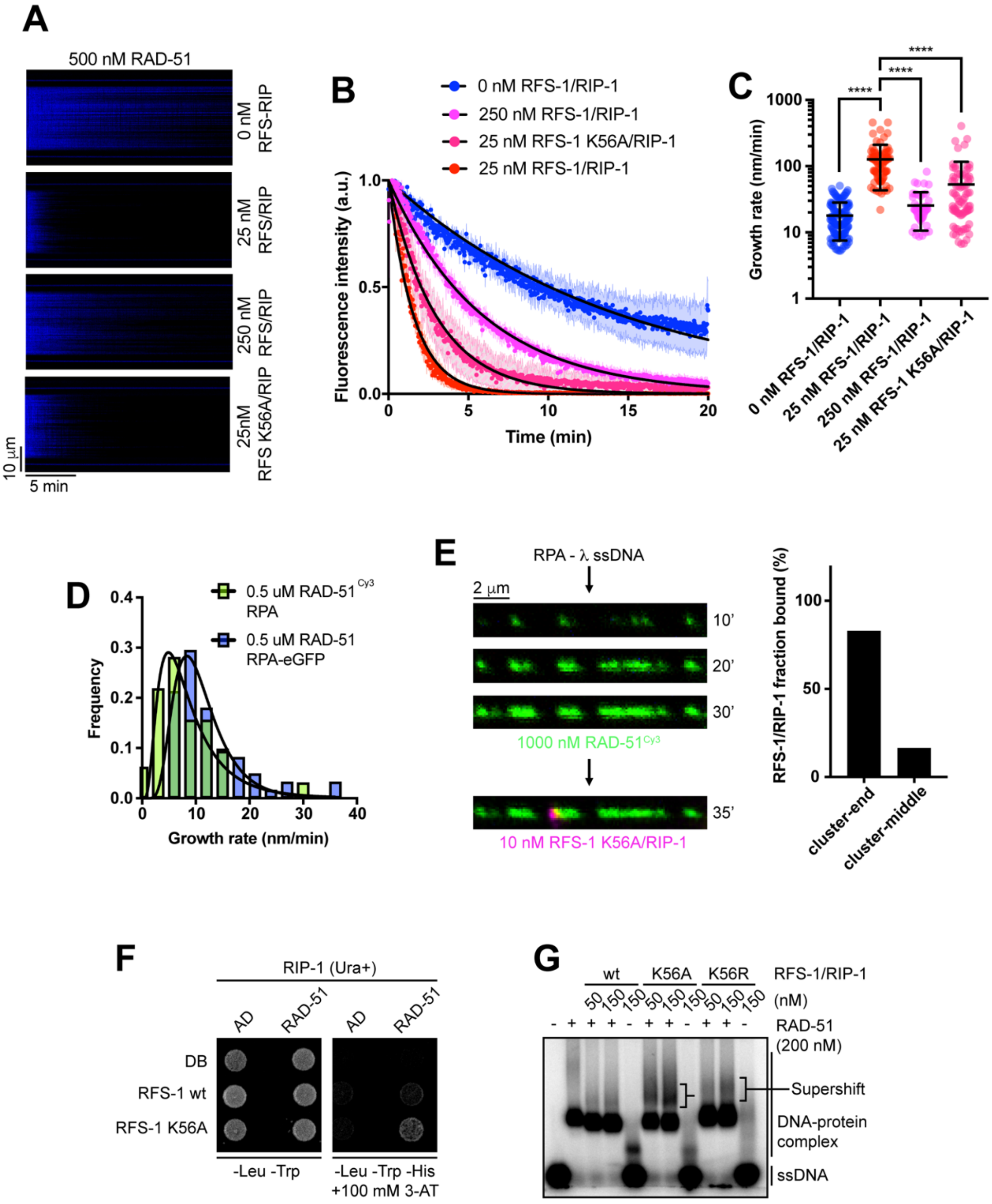
A
500 nM RAD-51
0 nM RFS-RIP
25 nM RFS/RIP
250 nM RFS/RIP
25nM RFS K56A/RIP
10 µm
5 min
B
0 nM RFS-1/RIP-1
250 nM RFS-1/RIP-1
25 nM RFS-1 K56A/RIP-1
25 nM RFS-1/RIP-1
Fluorescence intensity (a.u.)
Time (min)
C
Growth rate (nm/min)
****
0 nM RFS-1/RIP-1
25 nM RFS-1/RIP-1
250 nM RFS-1/RIP-1
25 nM RFS-1 K56A/RIP-1
D
0.5 uM RAD-51Cy3
RPA
0.5 uM RAD-51
RPA-eGFP
Frequency
Growth rate (nm/min)
E
RPA - λ ssDNA
2 µm
10'
20'
30'
1000 nM RAD-51Cy3
35'
10 nM RFS-1 K56A/RIP-1
RFS-1/RIP-1 fraction bound (%)
cluster-end
cluster-middle
F
RIP-1 (Ura+)
AD
RAD-51
DB
RFS-1 wt
RFS-1 K56A
-Leu -Trp
-Leu -Trp -His
+100 mM 3-AT
G
wt
K56A
K56R
RFS-1/RIP-1
(nM)
RAD-51
(200 nM)
Supershift
DNA-protein
complex
ssDNA

Figure S7. Extended analysis of RFS-1/RIP-1 Walker A mutants. Related to Fig. 5. (A) Kymograph showing the displacement of RPA-eGFP by RAD-51 in indicated conditions. **(B)** Normalized fluorescence intensity for RPA–eGFP signal in indicated conditions; shaded area represents SEM. (n = 3-7 molecules). Black lines represent exponential fits. **(C)** Quantification of growth rates in indicated conditions. $P < 0.0001$. Mann-Whitney test. **(D)** Growth rate distribution of unlabelled RAD-51 on RPA-eGFP coated ssDNA (n = 61 clusters; geometric mean of 10.1 ± 1.6 nm/min, 3 independent molecules) and growth rate distribution of RAD-51^{Cy3} on RPA coated ssDNA (n = 32 clusters, geometric mean of 7.5 ± 1.9 nm/min, 5 independent molecules). Lines represent lognormal fits. **(E)** Confocal fluorescence images taken after subsequent incubation-detection cycles using RPA-λ ssDNA and 1000 nM RAD-51^{Cy3} (green) or RFS-1 K56A/RIP-1(A647) (magenta). Cumulative incubation time is indicated. Representative images are shown. Quantification of filament end binding frequencies of RFS-1 K56A/RIP-1(A647) from two independent experiments. **(F)** DB-RFS-1 and AD-RAD-51 interaction in yeast two-hybrid, indicated by survival on media containing 3-aminotriazole (3-AT) in the absence of histidine. Growth on media lacking leucine and tryptophan is a positive control for plasmid transfection. RIP-1 was co-expressed in the same strains on a Ura-selectable plasmid. **(G)** EMSA showing formation of additional ssDNA-protein complexes (supershift) when RFS-1 K56A or RFS-1 K56R/RIP-1 variants were used. For these experiments, proteins were mixed together and incubated on ice for 5 min, 1 nM (in molecules) 60-mer radio-labelled ssDNA was added and incubated for 10 min at 25 °C with indicated proteins followed by 10 min crosslinking with 0.25% glutaraldehyde at 25 °C. Protein-DNA complexes were resolved using 1 % agarose gel electrophoresis at 4 °C for 2 h 20 min.

REAGENT or RESOURCE	SOURCE	IDENTIFIER
Oligonucleotides		
Cy5-90mer 5'-Cy5-AAATCAATCTAAAGTATATATGAGTAAACTTGGTCTGACAGTTACCAATGCTTAATCAGTGAGGCACCTATCTCAGCGATCTGTCTATTT-3'	Sigma Aldrich, This study	N/A
Cy5-49mer 5'-Cy5-AGCTACCATGCCTGCACGAATTAAGCAATTCGTAATCATGGTCATAGCT-3'	Sigma Aldrich, This study	N/A
FAM-49mer 5'-FAM-AGCTACCATGCCTGCACGAATTAAGCAATTCGTAATCATGGTCATAGCT-3'	Sigma Aldrich, This study	N/A
60mer 5'-ACGCTGCCGAATTCTACCAGTGCCTTGCTAGGACATCTTTGCCCACCTGCAGGTTCACCC-3'	Sigma Aldrich, This study	N/A
150mer 5'-TCTTATTTATGTCTCTTTTATTTCATTTCCTATATTTATTCCTATTATGTTTTATTCATTTACTTATTCTTTATGTTCATTTTTTATATCCTTTACTTTATTTTCTCTGTTTATTCATTTACTTATTTTGTATTATCCTTATCTTATTTA -3'	Sigma Aldrich, This study	N/A
FAM-40mer 5'-FAM-TAATACAAAATAAGTAAATGAATAAACAGAGAAAATAAAG-3'	Sigma Aldrich, This study	N/A
Complimentary 40mer 5'-CTTTATTTTCTCTGTTTATTCATTTACTTATTTTGTATTA -3'	Sigma Aldrich, This study	N/A
crRNA *rfs-1* K56 5'-TTTAGGAGTTGGTAAAACAC-3'	IDT, This study	N/A
crRNA *HA::AID*::*brc-2* 5'-TTTTTAGATGAGTCACCCAT -3'	IDT, This study	N/A
crRNA dpy-10 5'-GCTACCATAGGCACCACGAG-3'	IDT, This study	N/A
ssDNA repair template *rfs-1K56A* 5'-TTCATCCAGGAAAATGCTACGAAATTGATGGCGATCTGGGTGTAGGAGCTACGCAAGTATGAATTCATATATTTTATTTAGAGAATTTTCC-3'	IDT, This study	N/A
ssDNA repair template *rfs-1K56R* 5'-TTCATCCAGGAAAATGCTACGAAATTGATGGCGATCTGGGTGTAGGACGAACGCAAGTATGAATTCATATATTTTATTTAGAGAATTTTCC-3'	IDT, This study	N/A
ssDNA repair template HA::AID::brc-2 oligo 1 5'-CAGACTTTACCAGAATATTGTGACATCGACCGATGTACCCATACGATGTTCCAGATTACGCTATGCCTAAAGATCCAGCCAAACCTCCGGCCAAGGCACAAGTTGTGGGATGGCCACCGGTGAGATCATACCGGAA-3'	IDT, This study	N/A
ssDNA repair template HA::AID::brc-2 oligo 2 5'-GTTGTGGGATGGCCACCGGTGAGATCATACCGGAAGAACGTGATGGTTTCCTGCCAAAAATCAAGCGGTGGCCCGGAGGCGGCGGCGTTCGTGAAGGGTGACTCATCTAAAAAAGTGTTAGTCAAGATTTA-3'	IDT, This study	N/A

ssDNA repair template *dpy-10* 5'-CACTTGAACTTCAATACGGCAAGATGAGAATGACTGGAAACCGTACCGCATGCGGTGCCTATGGTAGCGGAGCTTCACATGGCTTCAGACCAACAGCCTAT-3'	IDT, This study	N/A
ssDNA lambda precursor oligo 1 5'-GGGCGGCGACCTGGACAA-3'	IDT, This study	N/A
ssDNA lambda precursor oligo 2 5'-AGGTCGCCGCCCTTTTTTTT(BT)TT(BT)TT(BT)-3'	IDT, This study	N/A
ssDNA lambda precursor oligo 2 5'-T(BT)TT(BT)TT(BT)TTTTTTTAGAGTACTGTACCTAGCATCAATCTTGTCC-3'	IDT, This study	N/A
lambda DNA hairpin oligo 1 5'-AGGTCGCCGCCCGGAGTTGAACGT(BT)T(BT)TT(BT)TT(BT)ACGTTCAACTCC-3'	IDT, This study	N/A
lambda DNA hairpin oligo 2 5'-GGGCGGCGACCTCAAGTTGGACAAT(BT)TT(BT)TT(BT)T(BT)TGTCCAACTTG-3'	IDT, This study	N/A
gDNA tracrRNA 5'-GGACAGCAUAGCAAGUUAAAAUAAGGCUAGUCCGUUAUCAACUUGAAAAAGUGGCACCGAGUCGGUGCUUUUU-3'	IDT, This study	N/A
Lambda 2 crRNA 5'-GUGAUAAGUGGAAUGCCAUGGUUUUAGGAGCUAUGCUGUUUUG-3'	IDT, This study	N/A
Lambda 4 crRNA 5'-CAGATATAGCCTGGTGGTTCGUUUUAGGAGCUAUGCUGUUUUG-3'	IDT, This study	N/A
Lambda 5 crRNA 5'-GGCAAUGCCGAUGGCGAUAGGUUUUAGGAGCUAUGCUGUUUUG-3'	IDT, This study	N/A

Table S1: Oligonucleotides used in the study. Related to STAR METHODS.
